# **Circulating tumor cell detection: A prospective comparison between CellSearch**® **and RareCyte**® **platforms in patients with progressive metastatic breast cancer**

**DOI:** 10.1007/s10549-022-06585-5

**Published:** 2022-04-09

**Authors:** Luc Dirix, Andy Buys, Steffy Oeyen, Dieter Peeters, Vincent Liègeois, Annemie Prové, Dieter Rondas, Liesbet Vervoort, Véronique Mariën, Steven Van Laere, Peter Vermeulen

**Affiliations:** 1 GZA Sint Augustinus, Oosterveldlaan 24, 2610 Antwerp, Belgium; 2 CellCarta, Sint-Bavostraat 78-80, 2610 Antwerp, Belgium

**Keywords:** Circulating tumor cells,, Metastatic breast cancer, CellSearch, RareCyte

## Abstract

**Purpose:**

Circulating tumor cells (CTCs) are prognostic in patients with breast cancer. Several technical platforms exist for their enumeration and characterization. Comparative studies between these platforms are scarce. The RareCyte CTC detection is theoretically more sensitive than the established CellSearch platform, which identifies only CTCs that express EpCAM and cytokeratin.

This study prospectively compares CTC enumeration in patients with breast cancer in a paired analysis using these two platforms. It investigates survival outcomes in groups defined by a CTC count threshold.

**Design:**

CTC enumeration was performed on 100 samples obtained from 86 patients with progressive metastatic breast cancer (MBC) in two independent laboratories each blinded to the clinical data and the results from the other platform.

**Results:**

One hundred paired samples were collected and CTC counts were determined using the CellSearch and RareCyte CTC platforms. In total, 65% and 75% of samples had at least one detectable CTC in 7.5 mL blood with the CellSearch and the RareCyte systems, respectively. CTC counts with the CellSearch system ranged from 0 to 2289 with a median of 3 CTCs, the RareCyte CTC counts ranged from 0 to 1676 with a median of 3 CTCs. The number of samples with 5 or more CTCs in 7.5 mL of blood (the poor prognosis cut-off validated with the CellSearch system) blood was 45% with the CellSearch test and 48% with the RareCyte test. CTC counts quantified with the CellSearch and the RareCyte systems were strongly correlated (Spearman’s *r* = 0.8235 (0.7450–0.8795) *p* < 0.001).

86 patients were included for Kaplan–Meier survival analysis. An increased mortality risk in patients with CellSearch of 5 CTCs or more per 7.5 mL blood, with a log-rank hazard ratio of 5.164 (2.579–10.34) (*p* < 0.001) was confirmed. The survival analysis with RareCyte CTC counts with the identical cut-off showed a significantly impaired survival with a hazard ratio of 4.213 (2.153–8.244) (*p* < 0.001).

**Conclusion:**

Our data demonstrate the analytical and prognostic equivalence of CellSearch and RareCyte CTC enumeration platforms in patients with MBC using the CellSearch cut-off. This is the first demonstration of prognostic significance using the RareCyte platform.

## Introduction

The concept of a liquid biopsy was introduced as a means of both avoiding tumor biopsies with the additional advantage of obtaining a more complete representation of spatial tumor heterogeneity [1]. Specifically, circulating tumor cells (CTCs) can be captured and studied in a non-invasive manner through simple blood draws. The ease of repetitive sampling also holds the promise of cataloging temporal tumor heterogeneity.

In addition to the biological study of CTCs, their enumeration has been shown to be prognostic for progression free (PFS) and overall survival (OS) in patients with metastatic breast cancer [2, 3]. More specifically, CTC counts can distinguish patients with relatively indolent disease (< 5 CTCs/7.5 mL) from patients with more aggressive disease (≥ 5 CTC/7.5 mL). The mere presence of CTCs after completion of locoregional and adjuvant treatment, predicts disease relapse in patients with early stage breast cancer (BC) [4].

A wide range of different methodologies is available for the isolation, enumeration, and characterization of CTCs. Our group has performed one of the few comparative studies for the enumeration of CTCs using three different platforms [5].

The CellSearch system is the only Food and Drug Administration (FDA)-cleared CTC detection technique. It isolates CTCs from whole blood by mixing it with ferrofluid consisting of magnetic particles coated with antibodies against epithelial cell adhesion molecule (EpCAM). After magnetic extraction of the CTCs, multiplex staining with immunofluorescently labeled monoclonal antibodies for epithelial cells (CK8/18/19), leukocytes (CD45), and a nuclear dye (DAPI) are applied. Finally, a semi-automatic fluorescence microscope visualizes the cells. CTCs are identified as nucleated cells expressing cytokeratins (CK) and EpCAM, lacking CD45 expression. CellSearch detected elevated numbers of CTCs, defined as 5 CTCs or more per 7.5 mL, is a significant negative prognostic factor for both PFS and OS [2].

The RareCyte CTC detection platform consists of the AccuCyte® sample preparation system and CyteFinder instrument. CTCs are isolated based on density using the AccuCyte kit and dedicated blood tubes and collector devices. First, nucleated blood cells are separated from the red blood cell fraction. Then they are spread on 8 standard glass slides that can be stained with a maximum of six fluorescent markers. For this study, a three-channel fluorescent staining kit was used for CD45, SYTOX orange (a nuclear dye), and a combined pancytokeratin and EpCAM antibody cocktail. These slides are then imaged and semi-automatically analyzed by the CyteFinder instrument, a digital scanning microscope. Theoretically, RareCyte has advantages over CellSearch in terms of CTC detection sensitivity. One major difference is that RareCyte does not employ a membrane marker for positive selection before sample visualization, as opposed to CellSearch which only enriches for EpCAM-positive cells. On the other hand, the reproducibility and clinical validity of CellSearch have been documented in several cancer types, while such data are sparse for RareCyte [7–10].

The aims of this study are to compare CTC counts as detected with the CellSearch and RareCyte platforms and assess survival outcomes in groups defined by a CTC threshold in a large prospective series of blood samples of patients suffering from progressive MBC. The subgroup of patients with triple negative breast cancer is of particular interest as it has been suggested that this subtype is characterized by a lower level of EpCAM expression and might therefore have CTCs that are detectable by the RareCyte platform that are not detected by CellSearch [11–13].

## Methodology

### Study design

Women with MBC starting a new line of treatment were enrolled at the Department of Medical Oncology, GZA Hospitals Sint-Augustinus, Antwerp, Belgium. Patients each donated paired samples of each 10 mL whole blood: one sample was analyzed with the CellSearch test and one with the RareCyte test. Patient recruitment started in February 2019 and ended in December 2020. Blood samples were collected at the Translational Cancer Research Unit (TCRU) and coded. Relevant clinical patient data were entered in a dedicated data base and updated every 3 months. Both CellSearch and RareCyte tests were performed according to standardized procedures. CellSearch tests were performed at the GZA Hospital, RareCyte tests were performed at CellCarta (Antwerp, Belgium) Both tests were performed and reported by operators and readers that have been trained and qualified by the manufacturer of the respective platforms. Investigators performing both tests were blinded to the clinical information of the patient. The results of the CellSearch test were not disclosed to the investigators performing the RareCyte test and vice versa. All clinical data were centrally collected.

### Description of clinical variables

Clinicopathological variables were entered in a dedicated database and included: age at diagnosis, age a sample collection, pathological breast cancer type, hormone receptor status, HER2 status based on an in situ hybridization test and the scored according to the ASCO-CAP guidelines, disease stage at initial diagnosis, disease free interval, extent of disease at time of blood draw, number of treatment regimens for advanced disease. Follow-up was updated every three months. The clinical follow-up was updated for this analysis until September 30, 2021.

### Study population

#### Inclusion and exclusion criteria

This study enrolled female patients with MBC starting a new or first line of systemic treatment. Patients were required to be at least 18 years old, to have a WHO performance status less or equal to 2 and to provide written informed consent.

### Measurement methods

#### CellSearch

For the CellSearch test, 10 mL of blood is collected in CellSave Preservative Tubes containing a cell preservative solution and is processed within 72 h. 7.5 mL of blood is transferred from the collection tube into a conical tube and admixed with 6.5 mL of buffer solution. This sample is then centrifuged to separate cellular blood components from plasma. The tube with separated plasma is placed in the CellTracks Autoprep system which detects and aspirates the plasma from the tube and expands the sample in buffer. Next, the system mixes the CTCs with ferrofluids coated with an antibody for the EpCAM molecule present on tumor cells of epithelial origin. The CTCs are then magnetically separated from other cellular components. The for EpCAM-positive CTCs enriched cell population is subsequently subjected to a staining procedure with DAPI and cytokeratin 8/18/19 and CD45 monoclonal antibodies for staining the nucleus, the cytoplasm of epithelial cells and that of co-enriched leukocytes, respectively. The sample is automatically transferred to a cartridge in a MagNest that is subsequently scanned on the CellTracks Analyzer II, a four-color semi-automated fluorescence microscope that captures image frames covering the entire surface of the cartridge for each of four fluorescent filter cubes. The main criteria for an object to be defined as a CTC include a round-to-oval morphology, a visible nucleus (DAPI), positive staining for CK8/18/19, and negative staining for CD45. All possible CTCs were verified by a trained reviewer. Positive control samples from the CellSearch CTC control kit were included in each run as per the manufacturer’s instruction.

#### RareCyte

For the RareCyte test, a blood sample is collected in a 10 mL AccuCyte® BCT tube (RareCyte, Seattle, USA). 7.5 mL of this blood is transferred into an AccuCyte® Separation Tube. Subsequently, the tube is centrifuged and four layers are identified: plasma on top, then a broad gray layer of platelets, a thin layer of white blood cells (buffy coat), and finally a layer of compacted red blood cells. After centrifugation, the Separation Tube is clamped to create a barrier between the red blood cells and the rest of the sample. Plasma is then removed and replaced with a high density fluid. The buffy coat is retrieved after a second centrifugation. Then, transfer fluid is added to the buffy coat and after 10–60 min of incubation, the sample is applied to charged microscope slides with a spreading device. After drying, slides are processed using the Ventana DISCOVERY ULTRA automated platform. This platform applies fluorescently labeled antibodies against pan-CK and EpCAM, CD45, and a nuclear dye. The stained slides are scanned on a CyteFinder® digital microscope and candidate CTCs are identified using CyteMapper® image analysis software. All CTCs are verified by a trained reviewer based on morphology and expression of both epithelial and nuclear stains without CD45 expression.

### Sample size

The sample size for this study was determined at 100 paired samples. To calculate the sample size for a study like this, data on marker prevalence, distribution of markers, error of measurement on the real outcome etc. are essential. Since these data are not available for the RareCyte test, assumptions were made to estimate an adequate sample size. The McNemar test was used to estimate the sample size needed to measure a given difference in positivity rate at a given cut-off point between the two CTC detection methods. Cut-off points for positivity for both tests were predefined at ≥ 1 CTCs/7.5 mL blood and at the clinically validated prognostic cut-off point for the CellSearch test of ≥ 5 CTCs/7.5 mL blood. Assuming an overall difference in positivity rate between both tests of 25%, a sample size of 85 samples offers a power of 80% to measure a two-sided difference of 15% in the proportion of samples testing positive in favor of one of both tests at a significance level of 5%.

### Statistics

Statistical analysis GraphPad Prism software version 9.2.0 (CA, USA) was used for primary statistical analysis and graph preparation. Continuous variables are described by median and range.

Normality of data was analyzed by D’Agostino-Pearson and the Kolmogorov–Smirnov test.

Overall survival was measured as days from study entry until death or censored at last follow-up.

Median OS was estimated by Kaplan–Meier survival curves. Survival curves were compared with a logrank test.

## Results

### Sample number and characteristics

**Fig. 1:**
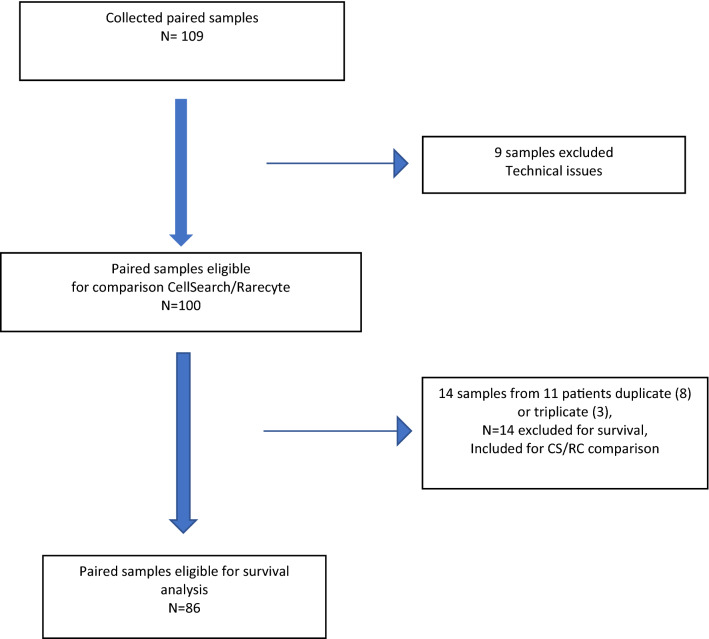
Schematic overview of patient and sample enrollment

One hundred and nine paired samples from 95 different patients with MBC were included in this study between February 2019 and December 2020. Nine paired samples were excluded because of wrong inclusion, practical problems or sample errors are shown in Fig. 1. The remaining 100 paired samples originated from 86 different patients. Eleven patients underwent two or more blood draws but did meet the inclusion criteria on each occasion. Survival analysis was performed on the 86 different patients, taking the first blood sample for the 11 patients with more than one collection. A CONSORT diagram summarizing patient inclusion and exclusion and sample collection is provided in Fig. 1.

### Patient characteristics

In total 86 patients with progressive disease were included in the analysis. The clinicopathological characteristics are summarized in Table 1. Median age at sample collection was 67 years (range 33–97). Fifty-seven patients (66%) were enrolled prior to the initiation of first-line treatment for advanced disease. No patients were lost to follow-up. By the time of this analysis (30-Sep-2021), 37 (43%) of the 86 patients have died of their disease. Follow-up ranges between 42.4 and 135.7 weeks.

**Table 1:** Patient demographics

Patients Number (N)	86	100%
Age (years) Median (Range)	67 y	(33–98)
Pathology		
IDA	65	(75%)
ILA	20	(23.5%)
NOS	1	(1.5%)
Molecular subtype		
ER + and/or PgR +	72	(84%)
HER2 +	12	(14%)
HER2 + /ER +	11	(12.7%)
HER2 + /ER-	1	(1.5%)
TN	13	(15%)
Lines of therapy		
First line	57	(66%)
Second line	6	(7%)
Third line	16	(19%)
Fourth line and beyond	7	(8%)
Sites of disease		
Bone	67	
Bone only	15	
Liver	16	
Lung	16	
Peritoneal/Pleural	17	
Brain and LMM	4	
Nodal		
Adrenal	1	

### Comparison between CTC count number with CellSearch and RareCyte

One hundred paired blood samples were collected and CTC counts were determined using the CellSearch and RareCyte system. In total, 65–75% of samples contained at least one detectable CTC in 7.5 ml blood with the CellSearch and the RareCyte system, respectively (Table 2). CTC counts with the CellSearch system ranged from 0 to 2289 with a median of 3 CTCs, the RareCyte CTC counts ranged from 0 to 1676 with a median of 3 CTCs. The number of samples meeting the CellSearch-validated prognostically relevant cut-off of 5 or more CTCs in 7.5 mL of blood was 45% with the CellSearch test and 48% with the RareCyte test.

**Table 2:** Distribution of categorical paired CTC counts

*N* = 100	CellSearch	RareCyte
Range CTC in 7.5 mL	0–2289	0–1676
Median CTC count	3	3
Mean CTC count	89	84

	CTC 0/7.5 mL	CTC 1/7.5 mL	CTC 2–4/7.5 mL	CTC ≥ 5/7.5 mL
CellSearch	35	6	14	45
RareCyte	25	9	20	46

	CellSearch			
RareCyte		< 5 CTC/7.5 mL	≥ 5 CTC /7.5 mL	Total
	< 5 CTC/ 7.5 mL	47	7	54
	≥ 5 CTC /7.5 mL	8	38	46
	Total	55	45	100

According to the Kolmogorov–Smirnov test and D’Agostino Pearson the CTC counts obtained with both the CellSearch and RareCyte were not normally distributed.

**Fig. 2:**
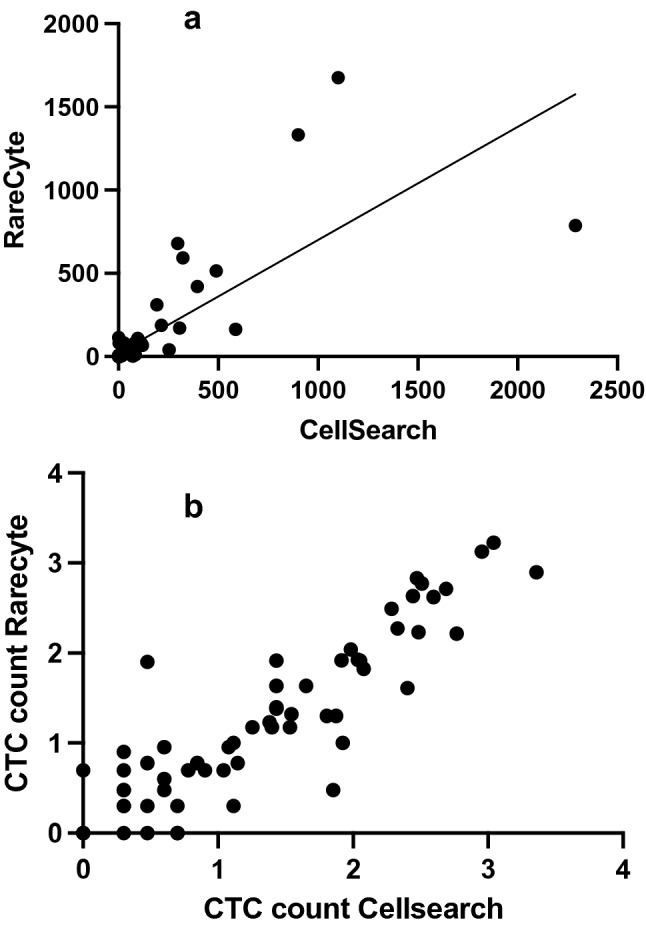
**A** Relationship CTC count CellSearch and RareCyte obtained from 100 samples and 86 patients with progressive metastatic breast cancer with Spearman *r* = 0.8179 (0.7378–0.8753)(*p* < 0.0001) **B** Relationship log CTC count CellSearch and log RareCyte

CTC counts quantified with the CellSearch and the RareCyte systems were strongly correlated (Spearman’s *r* = 0.823 (0.7443–0.8782) *p* < 0.001) (Fig. 2).

The Wilcoxon Signed Ranks Test did not show a significant difference between paired CTC counts with both tests (*r* = 0.83; *p* < 0.0001). The intraclass correlation coefficient showed a good intertest comparability (intraclass correlation coefficient *r* = 0.867; *p* < 0.001) (Table3).

**Table 3:** Numerical results of CTC count in 16 samples from 13 patients with TN MBC

CTC count CellSearch	CTC count RareCyte
35	21
3	2
24	17
2	2
3	1
2	2
45	43
0	0
2	0
27	24
0	0
1	0
2	3
214	187
490	514
96	109

### Comparison between CTC count with CellSearch and RareCyte in the triple negative cohort (*n* = 13)

**Fig. 3:**
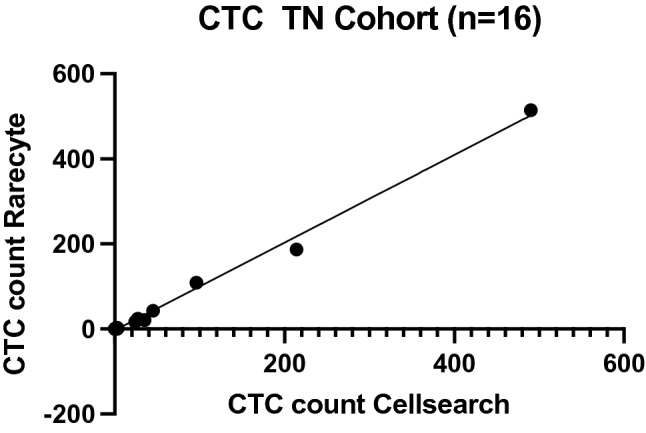
Correlation between CTC counts obtained from 16 samples of patients (*n* = 13) with progressive metastatic triple negative breast cancer

Sixteen paired samples obtained from 13 patients with triple negative MBC were available from this study. Three patients had a second sample collected at a point of further progression of disease. In total 946 CTCs were identified with CellSearch and 925 CTCs with RareCyte (Fig. 3, Table2). Both counts were highly correlated (*R*^2^ 0.9943 (*Y* = 1.031X-3.163)).

### Comparison between CTC count with CellSearch and RareCyte in the invasive lobular carcinoma cohort (*n* = 20)

In 20 samples from different patients, 4384 CTCs were counted with CellSearch and 3443 CTCs with RareCyte. The results were highly correlated with Spearman’s *r* of 0.9599. Similarly, in a rank test no difference was shown, between both counting methods.

### Comparison between CTC count ≥ 1 with CellSearch and RareCyte

A numerical difference in positive test results was observed between both systems using a ≥ 1 CTC cut-off (Table 2). With the RareCyte platform at least one CTC was detected in 10 samples in which the CellSearch platform did not, while the opposite was true in only 3 samples.

### Survival analysis

**Fig. 4:**
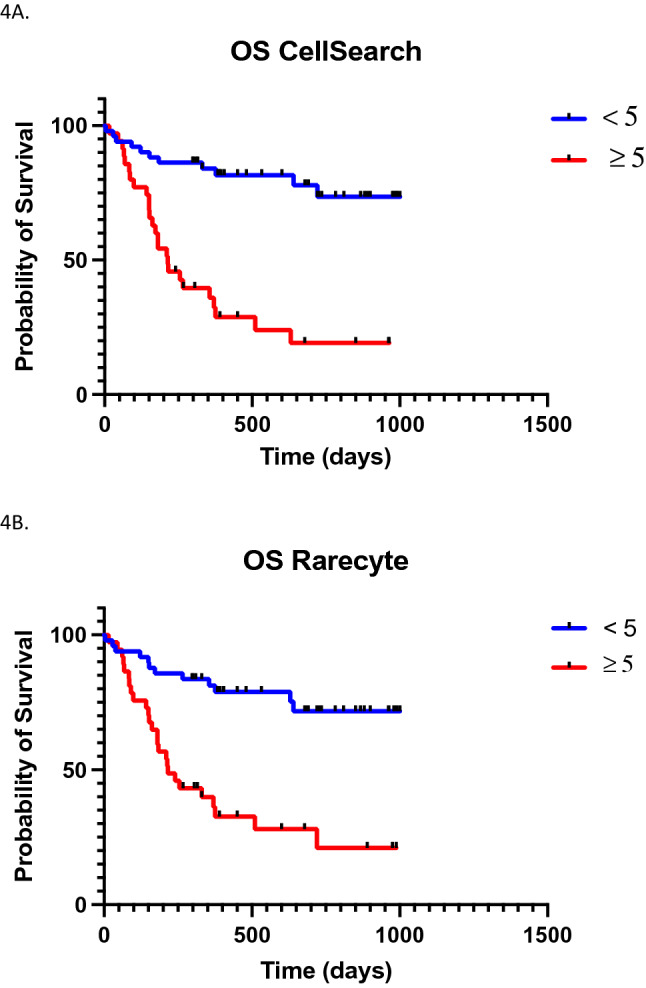
Overall Survival (days) in Patients with MBC for those with < 5 CTCs per 7.5 ml of whole blood and those in the group with ≥ 5 CTCs in 7.5 ml of whole blood (*n* = 86). **A** Cellsearch count with a log-rank HR 5.64 (2.579–10.34) (*p *< 0.0001). **B** RareCyte count with a log-rank HR 4.213 (2.153–8.244) (*p *< 0.0001)

All 86 patients were included for Kaplan–Meier survival analysis. If patients underwent multiple blood samplings, the survival analysis was based on their first CTC measurement. A significant increased mortality risk in patients with a CellSearch count of 5 CTCs or more per 7.5 mL blood was confirmed. The logrank hazard ratio was 5.164 (2.579–10.34) (*p* < 0.001) compared to the patients with less than 5 CTCs (Fig. 4A).

The survival analysis was repeated with the RareCyte CTC counts applying the identical cut-off. The patient survival again showed a significantly impaired survival with a hazard ratio of 4.213 (2.153–8.244) (*p* < 0.001) in patients with 5 CTCs or more per 7.5 mL blood (Fig. 4B).

## Discussion

The objectives of this study were to investigate whether there is a difference in the detection rate of two CTC counting methods, the CellSearch and RareCyte platforms, in blood samples of patients with progressive MBC and to assess survival outcomes in patient subsets defined by a CTC count threshold. We performed a comparison of both detection techniques in an unselected cohort of 109 blood samples, consecutively collected from 92 patients with progressive MBC patients before starting a new line of treatment. One hundred samples originating from 86 patients were evaluable for this analysis. It is of interest to point to the demographics of this cohort; nearly one in 4 patients suffered from lobular carcinoma and two out of three patients were unpretreated for advanced disease.

This is the first prospective double blinded comparison of these two platforms. In 100 evaluable paired blood samples from 86 patients, CTC counts of both platforms were highly correlated with overall good intertest comparability metrics. No significant differences were observed when CTC counts were compared as continuous variables or in test positive rates after dichotomization according to a cut-off of ≥ 5 CTCs per 7.5 mL blood. These findings indicate that the RareCyte system can be a valuable alternative to the FDA-cleared CellSearch system.

One paired sample showed a remarkable difference in CTC counts. In a 55-year-old patient with ER + /PR + /HER2 + lobular BC and metastatic disease to non-regional lymph nodes, 3 CTCs were counted with CellSearch and 80 with the RareCyte system. One explanation might be the existence of a single marker (EpCAM or CK) positive CTC population. Since both of these markers were present as one antibody cocktail in the RareCyte test, this hypothesis could not be further tested. Another explanation might be an unexpectedly high number of false positive CTCs detected by the RareCyte system. Future studies will be needed to assess whether there is a small subgroup of patients with MBC in whom higher numbers of EpCAM and/or cytokeratin single positive CTCs can be found.

We took a particular interest in 16 samples originating from 13 patients with triple negative MBC. This breast cancer subtype is suggested to be characterized by low EpCAM expression, which has led to numerous reports of a lower isolation performance of the CellSearch platform. In our previous work we failed to corroborate these reports [14]. We could not confirm the earlier suggestion that significantly more CTCs could be identified with RareCyte than with CellSearch platform [10]. Similarly, as in the overall study, no arguments for a superior sensitivity of RareCyte over CellSearch could be discerned. In fact, in this group the degree of nearly identical numerical results is striking (Table 2). It remains possible that there are few CTCs in our patient population with partial mesenchymal characteristics in which EpCAM is down regulated but cytokeratin remains detectable. Two-thirds of our population as a whole only received first-line chemotherapy. It is likely that more heavily treated cancers are more prone to mesenchymal transformation.

A numerical difference in positive test results was observed between both systems using a ≥ 1 CTC cut-off (Table 2). The RareCyte platform detected at least one CTC in 10 samples in which the CellSearch platform did not, the opposite was true in only 3 samples. Although this cut-off has been proven to be prognostically significant in studies in early BC, it remains to be established to what extent the ability to detect very low numbers of CTCs might be clinically valuable for molecular and genetic profiling of these cells. A possible explanation for the difference at low CTC count is that the RareCyte platform is in fact identifying cells that have a mesenchymal phenotype. However, the observed difference should be treated with caution, since the numeric CTC distribution in samples with very low CTC count is variable.

Currently the only clinically validated use of the CTC count in patients with MBC is the prognostic significance of the CTC enumeration. A survival analysis stratified by CTC counts dichotomized at ≥ 5CTCs/7.5 mL blood has been shown to be a reliable prognosticator for OS with the CellSearch platform. This was confirmed in our patient's cohort of 86 patients with the CellSearch platform and we have demonstrated for the first time that this is also true for the RareCyte platform. This observation corroborates our observation of the numerical correlation in the 100 samples in that CTC count by either platform can be used for enumeration and prognostication in patients with MBC using the ≥ 5 CTCs/7.5 mL threshold.

In summary, our data demonstrate the analytical and prognostic equivalence of CellSearch the RareCyte platform for those tumors with a putatively decreased EpCAM expression (i.e., triple negative subtype) was not observed. The numerical superiority of the RareCyte system in the very low CTC group (< or = 1) deserves further study. In-depth analysis controlling for actual EpCAM expression is planned.

## Acknowledgements

We would like to thank all patients for participating in the P1133 prospective comparative trial and RareCyte Inc. Seattle, US, for their unrestricted grant.

## Data Availability

Enquiries about data availability should be directed to the authors.

## References

[1] Alix-Panabières C, Pantel K (2021). Liquid Biopsy: from discovery to clinical application. Cancer Discov.

[2] Cristofanilli M, Budd GT, Ellis MJ, Stopeck A, Matera J, Miller MC (2004). Circulating tumor cells, disease progression, and survival in metastatic breast cancer. N Eng J Med.

[3] Bidard FC, Peeters DJ, Fehm T, Nole F, Gisbert-Criado R, Mavroudis D (2014). Clinical validity of circulating tumour cells in patients with metastatic breast cancer: a pooled analysis of individual patient data. Lancet Oncol.

[4] Janni WJ, Rack B, Terstappen LW, Pierga JY, Taran FA, Fehm T (2016). Pooled analysis of the prognostic relevance of circulating tumor cells in primary breast cancer. Clin Cancer Res.

[5] Van der Auwera I, Peeters D, Benoy IH, Elst HJ, Van Laere SJ, Prové A (2010). Circulating tumour cell detection: a direct comparison between the cell search system, the AdnaTest and CK-19/mammaglobin RT-PCR in patients with metastatic breast cancer. Brit J Cancer.

[6] Allard WJ, Matera J, Miller MC, Repollet M, Connelly MC, Rao C (2004). Tumor cells circulate in the peripheral blood of all major carcinomas but not in healthy subjects or patients with nonmalignant diseases. Clin Cancer Res.

[7] Kaldjian EP, Ramirez AB, Sun Y, Campton DE, Werbin JL, Varshavskaya P (2018). The RareCyte(R) platform for next-generation analysis of circulating tumor cells. Cytometry A.

[8] Campton DE, Ramirez AB, Nordberg JJ, Drovetto N, Clein AC, Varshavskaya P, Friemel BH, Quarre S, Breman A, Dorshner M, Blau S, Blau CA, Sabath DE, Stilwell JL, Kaldjian EP (2015). High-recovery visual identification and single-cell retrieval of circulating tumor cells for genomic analysis using a dual-technology platform integrated with automated immunofluorescence staining. BMC Cancer.

[9] Blau CA, Ramirez AB, Blau S, Pritchard CC, Dorschner MO (2016). A distributed network for intensive longitudinal monitoring in metastatic triple-negative breast cancer. J Natl Compr Canc Netw.

[10] Stilwell JL, Drovetto N, Ramirez AB, Campton D, Nordberg J, Varshavskaya P, et al. Abstract 1601: Clinical performance of the AccuCyte® - CyteFinder® System, a dual-technology platform for comprehensive collection and high-resolution imaging of circulating tumor cells. Cancer Research. 2015; 75 (15 Supplement):1601-.

[11] Sieuwerts AM, Kraan J, Bolt J, van der Spoel P, Elstrodt F, Schutte M (2009). Anti-epithelial cell adhesion molecule antibodies and the detection of circulating normal-like breast tumor cells. J Natl Cancer Inst.

[12] Van Laere SJ, Elst H, Peeters D, Benoy I, Vermeulen PB, Dirix LY (2009). Re: anti-epithelial cell adhesion molecule antibodies and the detection of circulating normal-like breast tumor cells. J Natl Cancer Inst.

[13] Paoletti C, Li Y, Muñiz MC, Kidwell KM, Aung K, Thomas DF (2015). Significance of circulating tumor cells in metastatic triple-negative breast cancer patients within a randomized, phase II trial: TBCRC 019. Clin Cancer Res.

[14] Peeters DJ, van Dam PJ, Van den Eynden GG, Rutten A, Wuyts H, Pouillon L (2014). Detection and prognostic significance of circulating tumour cells in patients with metastatic breast cancer according to immunohistochemical subtypes. Brit J Cancer.

